# Visualising associations between paired ‘omics’ data sets

**DOI:** 10.1186/1756-0381-5-19

**Published:** 2012-11-13

**Authors:** Ignacio González, Kim-Anh Lê Cao, Melissa J Davis, Sébastien Déjean

**Affiliations:** 1, Institut de Mathématiques - Université de Toulouse III et CNRS, UMR 5219, F-31062 Toulouse, France; 2 Queensland Facility for Advanced Bioinformatics and the Institute for Molecular Bioscience, The University of Queensland, 4072 St Lucia, QLD, Australia

## Abstract

**Background:**

Each omics platform is now able to generate a large amount of data. Genomics, proteomics, metabolomics, interactomics are compiled at an ever increasing pace and now form a core part of the fundamental systems biology framework. Recently, several integrative approaches have been proposed to extract meaningful information. However, these approaches lack of visualisation outputs to fully unravel the complex associations between different biological entities.

**Results:**

The multivariate statistical approaches ‘regularized Canonical Correlation Analysis’ and ‘sparse Partial Least Squares regression’ were recently developed to integrate two types of highly dimensional ‘omics’ data and to select relevant information. Using the results of these methods, we propose to revisit few graphical outputs to better understand the relationships between two ‘omics’ data and to better visualise the correlation structure between the different biological entities. These graphical outputs include Correlation Circle plots, Relevance Networks and Clustered Image Maps. We demonstrate the usefulness of such graphical outputs on several biological data sets and further assess their biological relevance using gene ontology analysis.

**Conclusions:**

Such graphical outputs are undoubtedly useful to aid the interpretation of these promising integrative analysis tools and will certainly help in addressing fundamental biological questions and understanding systems as a whole.

**Availability:**

The graphical tools described in this paper are implemented in the freely available R package mixOmics and in its associated web application.

## Introduction

‘Omics’ data now form a core part of systems biology by enabling researchers to understand the integrated functions of a living organism. However, the available abundance of such data (genomics, proteomics, metabolomics, interactomics...) is not a guarantee of obtaining useful information in the investigated system if the data are not properly processed and analyzed to highlight this useful information. A major challenge with the integration of omics data is therefore the extraction of discernable biological meaning from multiple omics data.

Recently, several authors have further improved statistical methodologies to integrate two highly dimensional data sets. Such methodologies include regularized and sparse variants of Canonical Correlation Analysis (CCA) [1-5] and Partial Least Squares (PLS) regression [6,7] - also referred as *projection-based methods*. These multivariate approaches aim at unravelling the correlation structure between two sets of data measured on the same samples. In addition, they achieve dimension reduction by summarizing the data into a small number of components or variates, which are linear combinations of the original variables. These exploratory approaches aim at exploiting coexpression between disparate types of biological measures instead of differential expression. The assumption relies on the fact that similar expression patterns across a set of samples are hypothesized to have a functional relationship [8]. In order to better understand the link between the different biological entities from highly dimensional data sets, several clustering techniques have been proposed in the literature. One category of approaches include simple criteria matching, which order the variables according to fold-change or univariate statistical tests for a given threshold. These variables are considered to be ‘clustered’ [9]. Other methods, such as self-organizing maps, use Euclidian distances. However, they are known to encounter difficulties in finding variables “negatively” (oppositely) associated with each other [10]. Another way is to comprehensively compare all variables against each other using a similarity measure, such as Pearson correlation coefficient [10,11], or mutual information [9]. Once these associations are graphically represented, the aim is to obtain fresh insights into the different biological functional levels, which then act as a foundation for new hypotheses.

So far, most the statistical integrative projection-based approaches cited above have been limited to numerical results, and little attention has been paid to either the interpretation of the results or the graphical outputs. In this article, we propose to revisit some graphical outputs mostly dedicated to exploratory approaches to highlight associations between two different types of biological entities. We have improved Correlation Circles plots, Relevance Networks and Clustered Image Maps (CIM) to be specifically adapted to the results of our previously published CCA or PLS methods [1,4,7]. These graphical outputs are implemented in the R package mixOmics^a^ that is dedicated to the integrative analysis of ‘omics’ data [12]. For users not familiar with the R programming language, a web application is also available at http://mixomics.qfab.org.

In the following ‘Background’ Section, we first describe the three graphical outputs used in mixOmics to visualise pair-wise associations between two types of biological variables. In the ‘Results and discussion’ Section, we assess the relevance of the proposed CIM and Relevance Networks on a simulation study. On two real data sets, we provide a thorough biological interpretation of the results obtained and compare the inferred statistical networks to known biological networks using data knowledge driven analyses. The ‘Methods’ Section describes how to compute the pair-wise similarity matrix to construct the graphical representations proposed.

## Background

We first briefly introduce PLS and CCA methodologies and their associated variants recently developed for the highly dimensional case. More details about the approaches are given in the ‘Methods’ Section. We review the three main graphical outputs proposed in mixOmics: Correlation Circle plots, Relevance Networks and Clustered Image Maps to visualise pair-wise associations between disparate biological entities highlighted by CCA or PLS.

### Integrative approaches

The two-block data matrices to be integrated are denoted *X*(*n*×*p*) and *Y*(*n*×*q*), where *p* and *q* are the total number of variables measured on the same *n* subjects. For example *X* is a gene expression matrix and *Y* contains metabolites concentrations, both transcripts and metabolites being measured on the same patients. CCA and PLS search for the largest correlation and covariance respectively between orthogonal components, also called *variates*, which are linear combinations of the *X* and *Y* variables. The number of chosen dimensions or components in CCA or PLS is *d*, with *d*≤ min(*p*,*q*) for CCA and *d*≤*p* for PLS.

In classical CCA and PLS regression, all variables from both data sets are included in the fitted linear combinations or variates. However, in the context of high throughput biological data, the number of variables often exceeds tens of thousands. In this case, linear combinations of the entire set of features make biological interpretability difficult as they contain too many variables to perform further tests or to generate biological hypotheses. Most importantly, the high dimensionality and the insufficient sample size lead to computational problems as CCA requires the computation of the inverse of the covariance matrices of *X* and *Y*. To circumvent this problem, regularized CCA (rCCA) has been recently proposed by [1] when dealing with ill-conditioned covariance matrices by adding a regularization term on their diagonal. Sparse PLS (sPLS) has been recently proposed to perform simultaneous variable selection in the two data sets [4,7]. sPLS includes Lasso penalization terms on the loading vectors (the vectors which weight are used in the determination of the PLS variates) to shrink some of the coefficients towards zero.

rCCA and sPLS are both implemented in the R package mixOmics[12]. These methodologies require to choose or tune the number of dimensions *d*, the regularization parameters for rCCA and the number of variables to select in both data sets for sPLS. Guidelines to choose these parameters are discussed in [1,7]. The biological relevancy of rCCA and sPLS has been recently demonstrated in several biological studies [13-18].

### Correlation Circle plots

Correlation Circle plots were primarily used for PCA outputs to visualise the relationship between variates and variables of the same type, where one single omics data set is analysed [19-23]. The use of such a graphical tool was then generalised to represent variables of two different types using statistical integrative approaches such as Canonical Correlation Analysis and Partial Least Squares regression [24].

**Figure 1:**
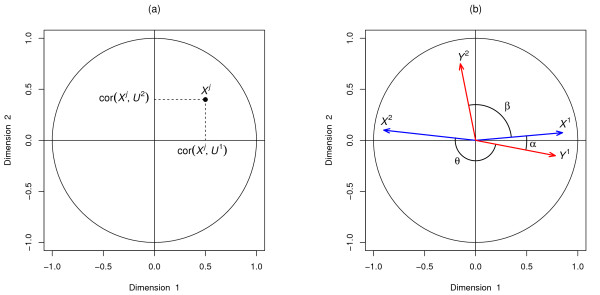
**Correlation Circle plot.a)** Coordinates of the *X*-variables on the plane defined by the first two variates *U*^1^ and *U*^2^. **b)** The correlation between two variables is positive if the angle is sharp cos(*α*)>0, negative if the angle is obtuse cos(*θ*)<0, and null if the vectors are perpendicular cos(*β*)≈0.

Although not very well known, this plot is an enlightening tool for data interpretation, as it enables a graphical examination of the relationships between variables and variates. In this plot, the coordinates of the variables are obtained by calculating the correlation between each original variable and their associated component (see Figure 1(a)). Because variables are usually centered and standardized, the correlation between each variable and a component is simply the projection of the variable on the axis defined by the component.

In this plot, variables can be represented as vectors (see Figure 1(b)) and the relationship (correlation) between the two types of variables can be approximated by the inner product between the associated vectors. The inner product is defined as the product of the two vectors lengths and their cosine angle. Thus, the nature of the correlation between two variables can be visualised through the angles between two vectors (Figure 1(b)): if the angle is sharp, the correlation is positive, if the angle is obtuse the correlation is negative and if the angle is right the correlation is null.

The centered and standardized variables are projected onto the space spanned by the two chosen components, inside a circle of radius 1. Thus, from the inner product definition, the longer the distance to the origin, the stronger the relationship between the variables. The variables closely located to the circumference of radius 1 can be directly interpreted, since the closeness on the plane corresponds to the closeness in the *d*-dimensional variables space.

**Figure 2:**
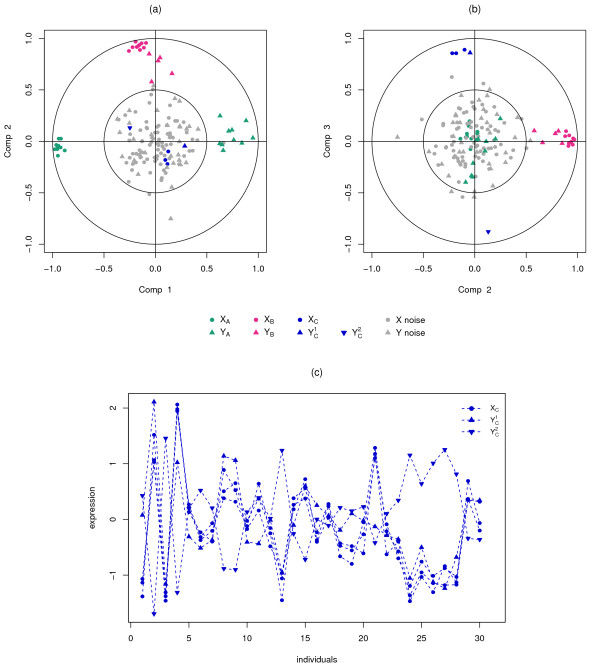
**Correlation Circle plots for the simulation study.** Correlation Circle plots for dimensions 1 and 2 **(a)**, and 2 and 3 **(b)**. The *X* and *Y* variables are represented by thick points and triangles respectively. The subsets of correlated variables are colored according to the legend. Expression profiles of some positively and negatively correlated variables across samples **(c)**.

For variables closely located to the origin, it means that some information can be carried on other axes and, it might be necessary to visualise the Correlation Circles plots in the subsequent dimensions (see example in Figure 2).

For the sake of interpretability, variables are not represented as vectors but as the end points of the vectors in mixOmics. Two circles are usually represented, of radii 0.5 and 1, to better visualise the ‘important’ variables. Figure 2 gives an example of the different scenarios that can be encountered when visualising the correlation structure between two data sets. The data come from the simulation study of the following ‘Results and discussion’ Section.

The variables or groups of variables strongly positively correlated are projected closely to each other on the Correlation Circle. This is the case, for instance, with *X*_*B*_ and *Y*_*B*_ on the dimension 2 (Figure 2(a-b)) and *X*_*C*_ and $Y_{C}^{1}$ on the dimension 3 (Figure 2(b)). When the correlation is strongly negative, the groups of variables are projected at diametrically opposite places on the Correlation Circle. This occurs, for instance, with *X*_*A*_ and *Y*_*A*_ on the dimension 1 (Figure 2(a)) and *X*_*C*_ and $Y_{C}^{2}$ on the dimension 3 (Figure 2(b)). The variables or groups of variables that are not correlated are situated 90° one from the other in the circle (for instance, *X*_*A*_ and *Y*_*B*_ (Figure 2(a)) and *X*_*C*_ and *Y*_*B*_ (Figure 2(b))).

Correlation Circle plots were found to supplement pair wise correlation approaches [25]. In the high dimensional case, the interpretation of the correlation structure between variables from two data sets can be difficult, and a threshold can be chosen to remove some weaker associations.

### Relevance networks

A conceptually simple approach for modelling net-like correlation structures between two data sets is to use Relevance Networks. This concept was introduced by [10] as a tool to study associations between pair of variables coming from several types of genomic data. This method generates a graph where nodes represent variables, and edges represent variable associations. The Relevance Network is built in a simple manner. First, the correlation matrix is inferred from the data. Second, for every estimated correlation coefficients exceeding (in absolute value) a prespecified threshold between two variables (say 0.6), an edge is drawn between these two variables, otherwise, no edge is drawn and these two variables are considered not associated for this threshold, and the variables/nodes with no link are not represented in the graph.

The construction of biological networks (gene-gene, protein-protein, etc.) with direct connections within a variable set is of considerable interest amongst biologists, and has been extensively used in the literature. Therefore, we will not consider this case and focus rather on the representation between variables of two different types. We will thus display Relevance Networks through the use of bipartite graph (or bigraph), where variables/nodes from *X* can only be connected to variables/nodes from *Y*.

Instead of computing the Pearson correlation coefficients between each pair of variables as was proposed by [10], bipartite networks are inferred using a pair-wise similarity matrix directly obtained from the outputs of the integrative approaches (regularized) CCA and (sparse) PLS. The values in the similarity matrix are computed as the correlation between the two types of projected variables onto the space spanned by the first components retained in the analysis. The values in the similarity matrix can be seen as a robust approximation of the Pearson correlation (see Section ‘Methods’).

The advantage of relevance networks is their ability to simultaneously represent positive and negative correlations, which are missed by methods using Euclidian distances or mutual information. Another advantage is their ability to represent genes in several pathways, and, most importantly for our purpose, to represent correlations across disparate biological measures. One of the main limitation of relevance networks is that it requires extensive computing ressources as mentioned by [26] to compute the comprehensive pair-wise associations when the underlying network is fully connected, i.e. when there is an edge between any pair of two types *X* and *Y* variables (see also [27] who recently proposed an R package for fast computation of the correlations). In the case of the sparse PLS, extensive computing is not required since the pair-wise association are only computed for the variables selected by the approach. A threshold is also proposed to remove some weaker associations.

Since the relevance networks are visual representations of the correlations between variables, one looks for clusters or sub-networks of subsets of variables, where the edge colors indicate the nature of the correlation (positive, negative, strong or weak). Each of these clusters often highlight a specific correlation structure between the features. More details about the relevance networks interpretation can be found in the Section ‘Results and discussion’.

### Clustered Image Maps

Clustered Image Maps (CIM), also called ‘clustered correlation’ or ‘heatmaps’ were first introduced by [11,28,29] to represent either the expression value of a single data set, or the Pearson correlation between two matched data sets [11,30]. This type of representation is based on a hierarchical clustering simultaneously operating on the rows and columns of a real-valued similarity matrix. This is graphically represented as a 2-dimensional coloured image, where each entry of the matrix is coloured on the basis of its value, and where the rows and columns are reordered according to the hierarchical clustering. Dendrograms (tree diagrams) illustrating the arrangement of the clusters produced by the hiearchical clustering are added to the left (or right) side and to the top (or bottom) of the image. The color in the heatmap indicates the nature of the correlation between subsets of variables (positive, negative, strong or weak), while the dendrogram indicate the proximity between correlated variables. In practice, one looks for well defined large rectangles or squares of the same color corresponding to long branches of the dendrograms. More details about the CIM interpretation can be found in the the Section ‘Results and discussion’.

The similarity matrix represented by the CIM is the same as in the relevance networks described above. CIM is a visualisation tool that complements well the Correlation Circles plots and the Relevance Networks as clusters of subsets of variables of the same type correlated with subsets of variables of the other type can be observed. This complementarity of three graphical outputs is illustrated in the Section ‘Results and discussion’ on the Nutrimouse case study.

### Implementation in mixOmics

Correlation Circles plots, Relevance Networks and Clustered Image Maps are implemented in the R package mixOmics[12] to be applied to a variety of integrative approaches implemented in the package, such as rCCA and sPLS methodologies. Full tutorials on how to analyse data sets with different methodologies and how to obtain specific graphical outputs with desired legends and colors are available on the website http://www.math.univ-toulouse.fr/~biostat/mixOmics. For users not familiar with the R programming language, an associated web application is available at http://mixomics.qfab.org and provides a Cytoscape plugin to display the Relevance Networks in an attractive manner.

## Results and discussion

We investigate the relevance of Correlation Circle plot, Relevance Networks and CIM representations, firstly on a simulated data set to assess if the proposed graphical outputs are able to highlight pair-wise association structure between two data sets, and secondly on two biological data sets to assess the biological relevance of such graphical tools.

### Simulated data

#### Data sets

We generated two data sets *X* and *Y* with an equal number of 30 observations in each data set. A subset of relevant variables in *X* were associated with a subset of relevant variables in *Y* according to the model described below, and the remaining variables were simulated as noise. This simulation study enables to assess if the proposed graphical representations allow differentiate the associated groups of relevant variables from the noisy variables. 

• The relevant *X* and *Y* variables were generated according to a normal distribution with zero mean and covariance matrix *Σ* defined by :

$$\Sigma =\left[\;\begin{array}{ll} \Sigma_{X\;X} & \;\Sigma_{{}_{X\;Y}}\\ \Sigma_{X\;Y}^{\prime } & \;\Sigma_{{}_{Y\;Y}} \end{array}\;\right],\;\text{with}\;\Sigma_{\text{XY}}=\left[\begin{array}{ll} A_{\text{XY}}\;0 & \;0\\ 0\;B_{\text{XY}} & \;0\\ 0\;0 & \;C_{\text{XY}} \end{array}\;\right].$$

• Details about the covariance matrices can be found in Additional file 1.

• *X* contains three independent sets of respectively 10, 10 and 3 cross-correlated variables: $X_{A}=\left[X_{A}^{1},\ldots ,X_{A}^{10}\right]$, $X_{B}=\left[X_{B}^{1},\ldots ,X_{B}^{10}\right]$ and $X_{C}=\left[X_{C}^{1},X_{C}^{2},X_{C}^{3}\right]$; and *Y* contains three independent sets of respectively 10, 5 and 2 cross-correlated variables: $Y_{A}=\left[Y_{A}^{1},\ldots ,Y_{A}^{10}\right]$, $Y_{B}=\left[Y_{B}^{1},\ldots ,Y_{B}^{5}\right]$ and $Y_{C}=\left[Y_{C}^{1},Y_{C}^{2}\right]$. These groups of variables are associated with each other according to the cross-correlation matrix *Σ*_*XY*_.

• The relevant variables in *X*_*A*_ and *Y*_*A*_ were generated with a negatively cross-correlation varying between −0.93 and −0.51. The variables in *X*_*B*_ and *Y*_*B*_ were generated with a positive cross-correlation varying between 0.5 and 0.85; and the variables in *X*_*C*_ and *Y*_*C*_ were generated with an absolute cross-correlation varying between 0.81 and 0.93, *X*_*C*_ is positively correlated with $Y_{C}^{1}$ and is negatively correlated with $Y_{C}^{2}$.

• The irrelevant (noisy) variables were simulated with a normal distribution with zero mean and covariance identity matrices and were added to the sets such that final data set contained 100 variables for *X* and 50 variables for *Y*. These variables are independent within the sets *X* and *Y* and with each other.

#### Analysis process

PLS canonical mode (*PLS-can*) was applied to these data sets and the graphical representations Correlation Circle plots, CIM and Relevance Networks resulting from the statistical approach were plotted. The first three dimensions were chosen for these graphical displays (correlation values between latent variables equal to 0.97, 0.94 and 0.95 respectively on each dimension, before decreasing for the following dimensions, Section ‘Methods’ gives the original references to the parameters tuning for the different approaches in mixOmics).

#### Graphical outputs

Figure 2 displays the corresponding correlation circle plots. Figure 2(a) highlights the strong negative correlation between clusters *X*_*A*_ and *Y*_*A*_ on dimension 1 and the strong positive correlation between clusters *X*_*B*_ and *Y*_*B*_ on dimension 2. Figure 2(b) underlines a strong positive correlation between *X*_*C*_ and $Y_{C}^{1}$ and a strong negative correlation between the latter and $Y_{C}^{2}$ on dimension 3. For that last case, Figure 2(c) represents the expression profiles of the variables across the 30 individuals and illustrates in a more intuitive manner the nature of the correlation between the variables.

**Figure 3:**
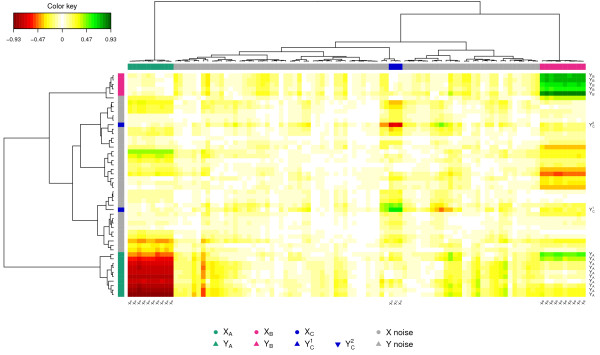
**CIM for the simulation study.** CIM on the simulated data with the PLS-can method. The green and red colours indicate positive and negative correlations respectively, whereas yellow indicate small correlation values. The clusters of variables are colored on the top and left side of the CIM as in Figure 2. The variables with blank names indicate variables with weak correlations (irrelevant variables).

The pair-wise similarity matrix was then computed using our proposed method (see Section ‘Methods’) for the first three PLS dimensions in order to display the CIM (Figure 3). The Euclidian distance and the Ward method were used for the hierarchical clustering. In the CIM display, each coloured block represents an association between subsets of the *X*-variables and the *Y*-variables. The green colour indicates that the *X* and *Y* clusters are positively correlated (cluster *X*_*B*_ and *Y*_*B*_, and cluster *X*_*C*_ and $Y_{C}^{1}$), and the red colour indicates a negative correlation in the *X*-*Y* cluster (cluster *X*_*A*_ and *Y*_*A*_, and cluster *X*_*C*_ and $Y_{C}^{2}$), whereas yellow indicate weaker correlation values. The dendrograms on the top and the left hand side of the map indicate how the clusters join, the longer the distance, the sharper the boundary between the coloured blocks.

The variables with blank names indicate variables with weak correlations (irrelevant variables). The CIM details in a more comprehensive manner than the correlation circle plots the correlations between all variables.

**Figure 4:**
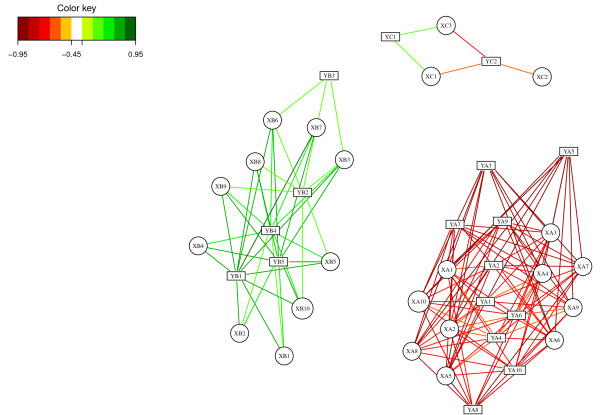
**Relevance Networks for the simulation study.** Relevance Networks obtained with sPLS-can on the simulated data using the network function in the mixOmics package. Green and red edges indicates positive and negative correlation respectively. *X* and *Y* variables are represented respectively as circles and rectangles.

The Relevance Networks obtained with PLS-can are displayed in Figure 4. Similarly to CIM representation, the pair-wise similarity matrix was computed for the first three dimensions (see Section ‘Methods’). Three relevant components were obtained setting a threshold to 0.5, linking the corresponding correlated subsets: *X*_*A*_ with *Y*_*A*_, *X*_*B*_ with *Y*_*B*_ and *X*_*C*_ with *Y*_*C*_. Note that none of the irrelevant variables were displayed in the network, demonstrating the good ability of the PLS approach to estimate the real simulated correlations.

#### Comparison with rCCA

The rCCA approach was also applied to these data sets with regularization parameters *λ*_1_=0.889 and *λ*_2_=0.889 for the first three dimensions (canonical values obtained were of 0.959, 0.925, and 0.881 on each dimension respectively, followed by much lower values). As expected, the graphical outputs were very similar to those with PLS-can.

In this simulation setting, accuracy of inferred networks from rCCA and PLS-can were similar, while networks inferred with Pearson correlation gave a much higher error rate, especially for a small number of samples (see Additional file 2).

This simulation study shows that Correlation Circle plots, Relevance Networks and CIM are able to highlight the relevant variables amongst the noisy ones and pinpoint the pair-wise association structure between the two data sets. In the following, we illustrate the use of such graphical outputs on real data sets and discuss the biological relevancy of the obtained results.

### Biological data

#### Data sets

These data sets are publicly available in the mixOmics package [12] and provide good illustrative examples for this Section. However, much larger biological data sets could be analysed through mixOmics as the integrative approaches rCCA and sPLS have been specifically developed to handle large data sets (several thousands of variables in both data sets).

**Nutrimouse data.** The data come from a nutrigenomic study [31] in which 40 mice from two genotypes (wild-type and PPAR *α* -/- deficient) were fed with five diets with contrasted fatty acid compositions. Oils used for experimental diets preparation were corn and colza oils (50/50) for a reference diet (REF), hydrogenated coconut oil for a saturated fatty acid diet (COC), sunflower oil for an Omega6 fatty acid rich diet (SUN), linseed oil for an Omega3 rich diet (LIN) and corn/colza/enriched fish oils (43/43/14) for the FISH diet. Expression of 120 genes in liver cells were acquired through microarray experiment and concentrations of 21 hepatic fatty acids were measured by gas chromatography. The study therefore includes two data matrices of size (40 × 120) for the gene expression and (40 × 21) for the fatty acids measurements.

**Liver toxicity data.** The data come from a liver toxicity study [32] in which 64 male rats of the inbred strain Fisher F344/N were exposed to low (50 mg/kg or 150 mg/kg) or to high (1500 mg/kg or 2000 mg/kg) doses of acetaminophen (paracetamol) in a controlled experiment. Necropsies were performed at 6, 18, 24 and 48 hours after exposure and the mRNA from the liver was extracted. Ten clinical chemistry measurements of variables containing markers for liver injury are available for each subject and the serum enzymes levels are numerically measured. The study therefore includes two data matrices of size (64 × 3116) for the gene expression and (64 × 10) for the clinical measurements.

#### Analysis process

Several methodologies are implemented in mixOmics to integrate data from two different types, to model the relationships between the two types of features in an appropriate manner (see Section ‘Methods’ for a brief description of the models). In the Nutrimouse data, we applied the methodology sPLS-can as the aim is to highlight highly correlated subsets of genes and hepatic fatty acids in the two data sets. This study was previously analysed with another approach (rCCA [1]). In this paper, the aim is to illustrate the usefulness of combining the three graphical outputs to interpret such results, and assessing the biological relevance of the Relevance Networks obtained.

In the Liver Toxicity data, we applied the methodology sPLS-reg as the aim is to highlight a subset of correlated genes which expression can predict the clinical chemistry measurements [33]. This analysis was performed in a previous paper to demonstrate the numerical good results of the sPLS-reg approach but no focus was made on the biological relevance of the results or on the use of variable graphical outputs. In this paper, we focus instead on the biological relevancy of the resulting Relevance Networks. In both studies, using these integrative methodologies and associated graphical outputs, the biological questions we ask are: which subsets of variables from both types are strongly positively or negatively correlated with each other? Do these selected features bring any relevant insight in relation to system under study?

Two parameters need to be tune in sPLS: the number of dimensions and the number of variables to select on each dimension. For both data sets, three dimensions were chosen (see numerical results presented in [1,7]). To illustrate the use of the proposed graphical outputs, we arbitrarily chose to select 50 transcripts or genes on each dimension. This rather large selection size (150 transcript or genes) is justified by the Gene Ontology (GO) analysis which require a sufficient number of variables to assess their biological relevance. The similarity matrices were computed from the sPLS method on the basis of the selected variables.

To highlight the strongest variable associations only, variables with an association score greater than 0.6 in absolute value were chosen to infer the Relevance Networks. This threshold was arbitrarily chosen in order to obtain biologically interpretable networks that were neither too sparse nor too dense. The obtained networks were then used as an input to Cytoscape [34] for visualization and GeneGo [35] and topGO [36,37] were used to assess the biological relevancy of the inferred associations between the different types of variables (see Additional file 3 for the R script used and how to export the network to a Cytoscape file format). This analysis is similar to the one performed by [38] who assessed the results of rCCA in a metabolic syndrome study. We then compared the obtained inferred networks to known biological networks through data driven and knowledge driven biological analyses.

#### Application to Nutrimouse data

**Figure 5:**
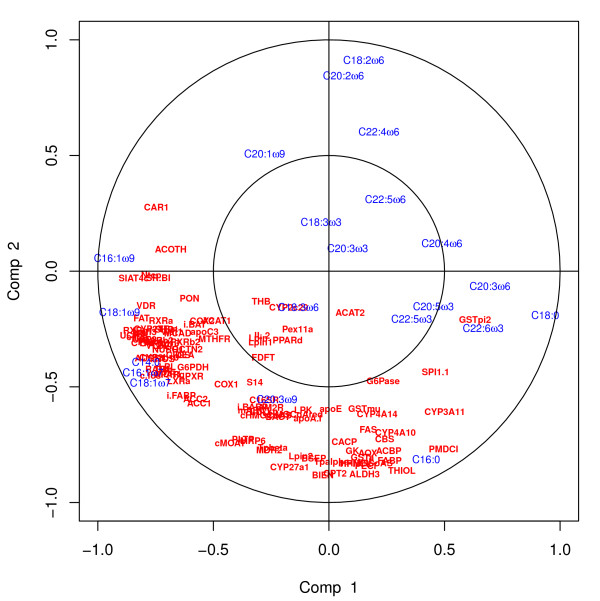
**Correlation Circle plots for the Nutrimouse study.** Correlation Circle plots for the first two sPLS dimensions (100 genes selected in total).

**Preliminary analysis comparing the different graphical outputs.** In order to illustrate the usefulness of the variable graphical outputs in a real case study, we first discuss the outputs obtained on the first two components, where 50 genes were selected on each dimension. The Correlation Circle plot (Figure 5) displays all fatty acids and the genes selected on each component (a 100 in total in this plot). It highlights subsets of variables that are important to define each component. For example C18:2 *ω*6, C20:2 *ω*6 and C16:0 are the fatty acids which variation mainly participate to the definition of the sPLS component 2 (top and bottom of the *y*-axis). Similarly, genes such as CAR1, ACOTH, SIAT4C, SR.BI, Ntop are positively correlated to each other, and to the fatty acid C16:1 *ω*9 and their variation participate to the definition of the sPLS component 1 (left-hand side of the *x*-axis).

**Figure 6:**
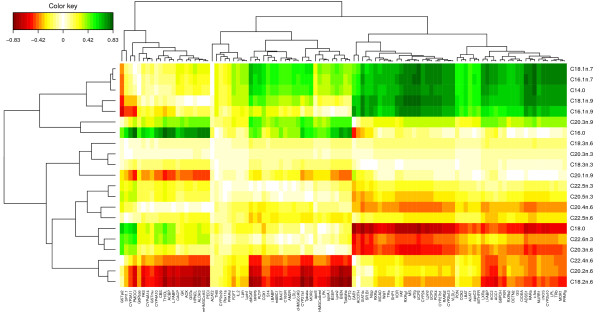
**CIM for the Nutrimouse study.** CIM for the first two sPLS dimensions (100 genes selected in total). Green (red) indicate high positive (negative) correlation.

While the CIM better highlights different clusters of variables and their degree of correlation (indicated by the colour code) than the Correlation Circle plots (Figure 6), the visualisation of the correlation within variables sets is more difficult to observe. For example, the Correlation Circle plot highlights a negative correlation between [C18:2 *ω*6, C20:2 *ω*6] and C16:0, which is less obvious in the CIM.

**Figure 7:**
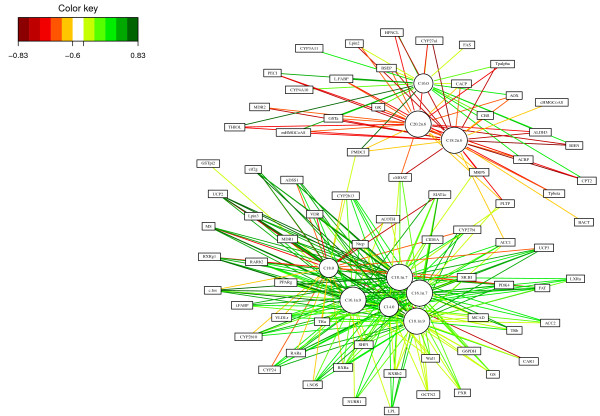
**Relevance Networks for the Nutrimouse study.** Relevance Networks obtained for the first two sPLS dimensions (100 genes selected in total). Green (red) indicate high positive (negative) correlation. Genes and fatty acids are represented respectively as rectangles and circles.

Finally, the relevance network representation (Figure 7) adds another layer of information as it allows the visualisation of variable groups in the network. In this case, the network highlights two main subsets of genes and fatty acids (top and bottom) which seem to contain very specific information in each of these groups. This information is slightly suggested on the CIM after a careful interpretation of the dendrograms, but is barely observable in the Correlation Circle plot. This comparison demonstrates the usefulness of such graphical outputs, as well as their complementarity to unravel the complex relationship structure between these different biological features. In the following, we discuss the biological relevance of the subsets of genes and fatty acids highlighted by the full sPLS-can analysis (with 3 sPLS components) using GeneGo and topGO. For an easier visualisation, the cytoscape software was used to represent the networks, but the similarity matrix was estimated with mixOmics.

**Figure 8:**
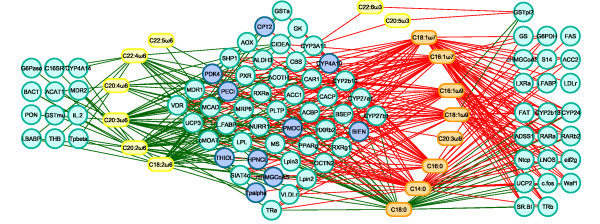
**Relevance Networks for the Nutrimouse study.** Relevance Networks generated with Cytoscape based on the proposed estimation of the pair-wise associations between seleted genes and fatty acids. Green and red edges indicate positive and negative correlation respectively.

**GeneGO analysis.** The Relevance Network generated for the Nutrimouse data at a threshold 0.6 highlighted two subsets of fatty acids, and three subsets of genes (Figure 8). Considering first the fatty acids, the yellow group on the left-hand side contained all the *ω*6 fatty acids from the data set (C18:2 *ω*6, C20:2 *ω*6, C20:4 *ω*6, C20:3 *ω*6, C22:5 *ω*6, and C22:4 *ω*6). The second group of fatty acids consisted of those in the *ω*9, *ω*7, and saturated fatty acid groups, along with the two *ω*3 fatty acids included in the data set. These groups made sense in the context of lipid biosynthetic pathways – one biosynthetic pathway leads to the production of *ω*6 lipids, while the *ω*9, *ω*7 and saturated lipids are the product of an alternative lipid biosynthetic pathway (orange nodes). The *ω*3 group was the exception in our analysis – it was generated by a pathway related to the *ω*6 pathway (yellow nodes), but based on the connectivity in our network, these fatty acids partitionned with the *ω*7, *ω*9 and saturated fatty acid group [39].

The three gene sets defined by network topology were: (1) a set of genes that were negatively correlated with only the *ω*6 lipid group; (2) a set of genes that were negatively correlated with the *ω*6 group, but largely positively correlated with the other lipid group; and (3) a gene set that was only associated with the second lipid group, with positive correlations to the *ω*3, *ω*7, *ω*9, and saturated fatty acids C14:0 and C16:0, but negatively correlated with the C18:0.

The *ω*6 group showed only negative correlations with genes selected by sPLS-can. This was consistent with the observations made by [31] that feeding mice a diet rich in *ω*6 fatty acids lead to the down regulation of several genes on the array.

The second group of genes contained many targets of PPAR *α*, a nuclear receptor transcription factor associated with the high-level regulation lipid metabolism (dark blue nodes). PPAR *α* targets are expected to be associated with long-chain polyunsaturated fatty acids from the *ω*3 family, while the final subset of genes involved in lipid biosynthesis is expected to be closely associated with the saturated and monosaturated fatty acids of the *ω*7 and *ω*9 families. Both of these associations were apparent in the network. An in-depth analysis of the Nutrimouse data is behind the scope of this article. The reader can refer to [31,39] for more details about the underlying biological interpretation.

#### Application to Liver Toxicity data

**Figure 9:**
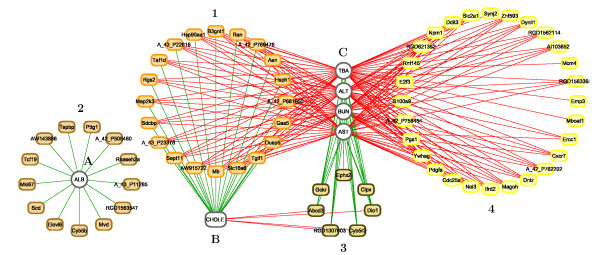
**Relevance Networks for the Liver Toxicity study.** Relevance Networks generated with Cytoscape based on the proposed estimation of the pair-wise associations between selected genes and clinical variables. Green and red edges indicate positive and negative correlation respectively. The network contained three groups of clinical chemistry measurements (white nodes): A [ALB], B [CHOLE] and C [ALT, AST, BUN, TBA] and four groups of genes (colored nodes) denoted 1, 2, 3 and 4.

**Visualization of the association between variables** Relevance Networks for the Liver Toxicity data were generated from the results obtained with the sPLS-reg method. The selected variables with a pair-wise association score greater than 0.6 in absolute value were used as an input to Cytoscape (Figure 9), green (red) edge color represent a positive (negative) correlation. This network contained three groups of clinical chemistry measurements (white nodes) denoted A, B and C and four groups of genes (colored nodes) denoted 1, 2, 3 and 4. Considering first the chemistry measurements, groups A and B only consisted of albumin [ALB] and cholesterol [CHOLE] levels respectively. Group C contained indicators of liver injury (Alanine Aminotransferase [ALT] and Aspartate aminotransferase [AST]), indication of renal injury (urea nitrogen [BUN]), and assessment of cholestasis – bile flow interruption (total bile acids [TBA]).

The four gene subsets defined by network topology (Figure 9) were: group 1: a set of genes that were positively correlated with the cholesterol levels B but negatively correlated with C; group 2, a set of genes that were negatively correlated with ALB levels only (group A); group 3, a set of genes positively correlated with group C and negatively correlated with group B; and group 4 a gene set with only positive correlations with group C.

**Figure 10:**
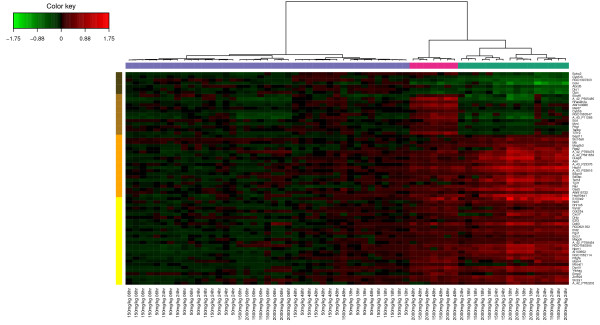
**Hierarchical clustering of the selected genes for the Liver Toxicity study.** Hierarchical clustering of the biological samples using the extracted genes from sPLS-reg network. Agglomerative hierarchical clustering was derived using the Euclidean distance as the similarity measure and Ward methodology. The resulting heatmap contains the genes in rows and samples in columns with red indicating up regulation, green down regulation and black no change. On the top of the heatmap, clusters of the biological samples are colored in violet, cyan and magenta for no, moderate or severe necrosis respectively. On the left hand side of the heatmap, gene clusters are shown (dark brown, brown, yellow and orange).

**Biological relevance of the extracted genes.** Hierarchical clustering (heatmap) of the biological samples on the extracted genes is displayed in Figure 10. This clustering highlights the groups of rats which were treated with different doses of acetaminophen (also found in [32]). Clusters labelled (coloured at the top of the heatmap) with either no (violet), moderate (cyan) or severe (magenta) necrosis of the centrilobular region of the rat liver were obtained by using the expression values of the genes extracted from the network. Levels of the clinical chemistry measurements on each group of samples are given in Additional file 4. Figure 10 also highlights the differences in gene expression profiles between each gene cluster (coloured in dark brown, brown, orange and yellow at the left side of the heatmap). Gene expression differences are clearly observed between the clusters.

Note that this heatmap represents the relationship between the samples (the rats) and the variables (gene expression) and the color inside the heatmap indicates the expression values of the genes, whereas the CIM represents the relationship between two groups of variables (clinical variables and gene expression) and the color inside the heatmap indicates the correlation between the variables.

The extracted genes were uploaded into topGO [36,37]. A Gene Ontology (GO) enrichment analysis from the gene list was then performed. GO terms significantly enriched include biological processes related to nitric oxide metabolism and cellular stress responses, including responses to unfolded proteins. The top GO molecular functions enriched in the gene set relate to protein binding, nucleotide binding, and enzyme activity (eg. hydrolase, phosphatase, decarboxylase). Cellular component GO terms enriched in the set mostly relate to very general locations, however both an endopeptidase complex and the peroxisome are also present in the list, reinforcing the association of the selected gene products with proteolysis and the response to stress and unfolded proteins.

The individual gene clusters in the sPLS-reg network (Figure 9) may also be examined for GO enrichment, as we have done for the larger cluster 4. For example, while examining the biological process terms associated this cluster, we saw an enrichment for processes involving xenobiotic transport, and interesting functional enrichments such as positive regulation of mesenchymal cell proliferation, a process that was previously observed to occur in other tissues in response to epithelial damage signalling to the underlying mesenchyme to initiate proliferation and tissue remodelling [40], and negative regulation of CREB transcription factor activity, interesting due to the previous association of CREB transcription factor with responses to cytotoxic stress [41,42], particularly in renal tubular cells [43].

Analysis of the gene list using the GeneGo [35] network analysis algorithm identified a total of 14 networks with a significant enrichment of genes in the Relevance Network. The top five networks were (i) regulation of programmed cell death in response to stress; (ii) cell cycle and regulation of metabolism; (iii) cholesterol and sterol metabolism; (iv) regulation of programmed cell death in response to organic substances; (v) response to stress and presentation of endogenous antigens. A summary of these networks can be found in Additional file 5.

## Conclusions

Since several methodologies have been recently proposed to jointly analyse two data sets, the development or the improvement of graphical tools is now crucial to better visualise and understand complex associations between biological entities. In the omics era in particular, the deluge of data can make the interpretation of the results extremely difficult. In our R package mixOmics, we have proposed such graphical tools to ease the interpretation of the implemented integrative methodologies dedicated to the analysis of large biological data sets.

In this paper, we revisited and further developed three types of graphical displays to better understand and interpret the results obtained with CCA and PLS related methods. We thoroughly described how to interpret Correlation Circles plots, which are very insightful graphical outputs to represent the associations between two types of variables. To complement the Correlation Circle plots, we proposed two types of graphical displays: both CIM and Relevance Networks are graphical representations of a pair-wise similarity matrix directly estimated from the results of the integrative methodologies implemented in mixOmics. The results obtained on simulated and real data sets illustrated very well the usefulness of these graphical tools to further explore the relationships between two omics data sets. The thorough biological interpretation of the obtained inferred networks using geneGO analysis demonstrated the relevancy of the approach.

Full tutorials are available on http://www.math.univ-toulouse.fr/~biostat/mixOmics to use all the methodologies and graphical outputs implemented in mixOmics. An associated web application is also available at http://mixomics.qfab.org and provides a Cytoscape plugin to display the Relevance Networks in an attractive manner.

## Methods

We revisit and further develop graphical outputs to visualise correlation structures between two data sets. Correlation Circle plots, CIM and Relevance Networks all use as input by-products of the integrative approaches implemented in the mixOmics package. Both CIM and the Relevance Networks require the estimation of large scale association or pair-wise similarity matrix *M* as an input. Previously, several similarity measures have been proposed, including Pearson correlation coefficient [10,29,44,45], entropy and mutual information [9]. We propose instead a novel approach to estimate a pair-wise similarity matrix using the results of either PLS or CCA approaches.

We briefly describe the PLS and CCA methodologies and associated variants recently developed for the highly dimensional case, more details about these approaches can be found in [1,7]. We then describe how to estimate the pair-wise similarity matrix to construct Relevance Networks and CIM.

### CCA and PLS based methods

#### Notations

We focus on two-block data matrices denoted *X*(*n*×*p*) and *Y*(*n*×*q*) where the *p* variables *X*^*j*^ and *q* variables *Y*^*k*^ are of two types and are measured on the same samples or observations *n*, for j=1,…,p and k=1,…,q. We adopt the following notation: $M_{k}^{j}$ represents the element of the *k*th row and *j*th column of the matrix *M*.

#### CCA

CCA [46] looks for the largest correlation between a linear combination of the variables in the first set *X* and a linear combination of the variables in the second set *Y*. The first pair maximizes the correlation *ρ*_1_=cor(*Xa*^1^,*Yb*^1^) subject to var(*Xa*^1^)=var(*Yb*^1^)=1. The subsequent pairs (*Xa*^*l*^,*Yb*^*l*^), (*l*=2,…, min(*p*,*q*)) maximize the residual correlation with the additional requirements that each pair is to be uncorrelated with the previous pairs. In the following, we will refer to *a*^*l*^ and *b*^*l*^ as the *canonical loadings* (or weights). The resulting variables *U*^*l*^=*Xa*^*l*^ and *V*^*l*^=*Yb*^*l*^ are called the *canonical variates* and *ρ*_*l*_ are known as the *canonical correlations*.

#### PLS

PLS [47] searches for the largest covariance between linear combinations of the *X* and the *Y* variable. The first pair maximises cov(*Xa*^1^,*Yb*^1^) subject to ||*a*^1^||=||*b*^1^||=1. Similar to CCA, the subsequent pairs (*Xa*^*l*^,*Yb*^*l*^), (*l*=2,…,*q*) maximize the residual covariance with the additional requirements that each pair is to be uncorrelated with the previous pairs. The algorithm of PLS differ from CCA as it is solved in an iterative manner by sequentially decomposing the data matrices. The *a*^*l*^ and *b*^*l*^ are referred to *loading vectors* (or weights) and the linear combinations *U*^*l*^=*Xa*^*l*^ and *V*^*l*^=*Yb*^*l*^ as the *latent variables* (or variates). Several PLS algorithms have been proposed in the literature, for different shapes of data (SIMPLS [48], PLS1 and PLS2 [47], PLS-SVD [49]), as well as for different modelling aims (predictive like PLS2, or modelling like PLS-mode A, see [2,23,50]). In the present paper, we will refer to a PLS approach with two different aims. *PLS-reg* (for PLS-regression mode) is used to model an ‘asymmetric’ or uni-directional relationship between the two data sets, i.e. we want to predict the matrix *Y* with the data *X*. In that case, the model is *Y*=*AX* where *A* is the matrix of the regression coefficients. *PLS-can* (for PLS-canonical mode) is used to model an ‘symmetric’ way and therefore models a bi-directional relationship. In that case, we would like to model *AY*=*BX* where *A* and *B* are the matrices of the regression coefficients. PLS-can and CCA have very similar purposes.

#### Regularized and sparse based methods

**rCCA.** The high dimensionality and the insufficient sample size lead to computational problems as CCA requires the computation of the inverse of matrices *X*^′^*X* and *Y*^′^*Y*. To circumvent this problem, [1] developed a regularized (or ridge) extension of CCA (rCCA). rCCA solves the instability of the loadings due to multicollinearity by adding a regularization term on the diagonal of the ill-conditionned matrices, i.e. the covariance matrices. Thus, highly correlated variables get similar loadings, resulting in a grouping effect. The regularization terms *λ*_1_ and *λ*_2_ associated to each data set are chosen by cross-validation in order to maximize the first canonical correlation.

**sPLS.** Several sparse PLS have been proposed in the literature to select variables [6,7]. These approaches introduce *l*_1_ (Lasso) penalization terms on the loading vectors to shrink some of the coefficients towards zero, thus allowing for simultaneous variables selection in the two data sets. The sparse PLS therefore solves the problem of interpretability by selecting variables from both sets and therefore providing sparse sets of associated variables. In the article, we consider the sparse PLS proposed by [7] since both regression (sPLS-reg) and canonical mode (sPLS-can, [4]) are available. For practical purposes, the two penalization parameters associated to each data set were replaced by the number of variable to select on each data set and on each sPLS dimension.

**Parameters tuning.** Both rCCA and sPLS are implemented in mixOmics. These approaches require to choose the number of dimensions *d* and the regularization/penalization parameters associated to *X* and *Y*. For rCCA, the choice of these parameters is based on cross-validation (see [1] for more details). For sPLS, depending on the modes, several criteria are available to choose these parameters. They are based on the *Q*^2^ criterion for the regression mode, or on the maximisation of the correlation for the canonical mode (as was also proposed by [2,5], see the original article [7] for more details).

### Pair-wise variable associations for CCA

The similarity measure that we propose to use is analogous to a correlation coefficient. Firstly, similar to a Correlation Circle output, the *X*^*j*^ and *Y*^*k*^ variables are projected onto a low dimensional space. Let *d*≤ min(*p*,*q*) the chosen dimensions to adequately account for the data association, and let *Z*^*l*^=*U*^*l*^+*V*^*l*^ the equiangular vector between the canonical variates *U*^*l*^ and *V*^*l*^ (*l*=1,…,*d*). The coordinates of the variable *X*^*j*^ and *Y*^*k*^ are obtained by projecting them on the axes defined by *Z*^*l*^. The projection on the *Z* axes seems the most natural as *X* and *Y* are symmetrically analysed in CCA. Furthermore, [22] showed that the *Z* variables have the property to be the closest to *X* and *Y*, i.e. the sum of their squared multiple correlation coefficients with *X* and with *Y* is maximal.

Let $x^{j}={\left(x_{1}^{j},\ldots ,x_{d}^{j}\right)}^{\prime }$ and $y^{k}={\left(y_{1}^{k},\ldots ,y_{d}^{k}\right)}^{\prime }$ the coordinates of the variable *X*^*j*^ and *Y*^*k*^ respectively on the axes defined by *Z*^1^,…,*Z*^*d*^. These coordinates are obtained by computing the scalar inner product $x_{l}^{j}=\langle X^{j},Z^{l}\rangle$ and $y_{l}^{k}=\langle Y^{k},Z^{l}\rangle$ (*l*=1,…,*d*). As the variables *X*^*j*^ and *Y*^*k*^ are assumed to be of unit variance, the inner product is equal to the correlation between the variables *X* (or *Y*) and *Z*: $x_{l}^{j}=\text{cor}(X^{j},Z^{l})$ and $y_{l}^{k}=\text{cor}(Y^{k},Z^{l})$.

Then, for any two variables *X*^*j*^ and *Y*^*k*^, a similarity score can be computed as follows:

(1) $$M_{k}^{j}=\langle x^{j},y^{k}\rangle ={\left(x^{j}\right)}^{\prime }y^{k}\;$$

where $0\leq |M_{j}^{k}|\leq 1$. The matrix *M* can be factorized as *M*=**xy**^′^ with **x** and **y** matrices of order (*p*×*d*) and (*q*×*d*) respectively. When *d*=2, *M* is represented in the Correlation Circle by plotting the rows of **x** and the rows of **y** as vectors in a 2-dimensional Cartesian coordinate system. Therefore, the inner product of the *X*^*j*^ and *Y*^*k*^ coordinates is an approximation of their association score.

### Pair-wise variable associations for PLS

For PLS-reg, the association score $M_{k}^{j}$ between the variables *X*^*j*^ and *Y*^*k*^ can be obtained from an approximation of their correlation coefficient. Let *r* the rank of the matrix *X*, according to [51], PLS-reg allows for the decomposition of *X* and *Y* by:

(2) $$X=U^{1}{\left(\phi^{1}\right)}^{\prime }+U^{2}{\left(\phi^{2}\right)}^{\prime }+\cdots +U^{r}{\left(\phi^{r}\right)}^{\prime }$$

(3) $$Y=U^{1}{\left(\varphi^{1}\right)}^{\prime }+U^{2}{\left(\varphi^{2}\right)}^{\prime }+\cdots +U^{r}{\left(\varphi^{r}\right)}^{\prime }+E^{\left(r\right)}$$

where *ϕ*^*l*^ and *φ*^*l*^, are the regression coefficients on the variates *U*^1^,…,*U*^*r*^, and *E*^(*r*)^ is the residual matrix (*l*=1,…,*r*). By denoting *u*_*l*_ the standard deviation of *U*^*l*^, using the orthogonal properties of the variates and the decompositions in (2) and (3), we obtain $x_{l}^{j}=\text{cor}(X^{j},U^{l})=u_{l}\phi_{j}^{l}$ and $y_{l}^{k}=\text{cor}(Y^{k},U^{l})=u_{l}\varphi_{k}^{l}$. Let *d*<*r* the number of components selected to adequately account for the variable association, then for any two variables *X*^*j*^ and *Y*^*k*^, the similarity score is defined by:

(4) $$M_{k}^{j}=\langle x^{j},y^{k}\rangle =\overset{d}{\underset{l=1}{\sum }}x_{l}^{j}y_{l}^{k}=\overset{d}{\underset{l=1}{\sum }}u_{l}^{2}\phi_{j}^{l}\varphi_{k}^{l}\approx \text{cor}(X^{j},Y^{k})\;,$$

where $x^{j}={\left(x_{1}^{j},\ldots ,x_{d}^{j}\right)}^{\prime }$ and $y^{k}={\left(y_{1}^{k},\ldots ,y_{d}^{k}\right)}^{\prime }$ are the coordinates of the variable *X*^*j*^ and *Y*^*k*^ respectively on the axes defined by *U*^1^,…,*U*^*d*^. When *d*=2, a Correlation Circle representation is obtained by plotting **x**^*j*^ and **x**^*k*^ as points in a 2-dimensional Cartesian coordinate system.

For PLS-can, the association score $M_{k}^{j}$ is calculated by substituting $y_{l}^{k}=\text{cor}(Y^{k},V^{l})$ in (4) for *l*=1,…,*d*, as in this case the decomposition of *Y* is given by:

$$Y=V^{1}{\left(\varphi^{1}\right)}^{\prime }+V^{2}{\left(\varphi^{2}\right)}^{\prime }+\cdots +V^{r}{\left(\varphi^{r}\right)}^{\prime }+E^{\left(r\right)}$$

where *φ*^*l*^ (*l*=1,…,*r*), are the regression coefficients on the variates *V*^1^,…,*V*^*r*^. Then,

$$M_{k}^{j}=\langle x^{j},y^{k}\rangle =\overset{d}{\underset{l=1}{\sum }}u_{l}^{2}\sigma_{l}^{2}\phi_{j}^{l}\varphi_{k}^{l}\approx \text{cor}(X^{j},Y^{k})\;,$$

where $\sigma_{l}^{2}$ is the variance of *V*^*l*^.

### Constructing Relevance Networks

Bipartite networks are inferred using the pair-wise similarity matrix *M* defined in (1) and (4) for (r)CCA and (s)PLS results respectively. Entry $M_{k}^{j}$ in the matrix *M* represents the association score between *X*^*j*^ and *Y*^*k*^ variables. Then, by setting a user-defined score threshold, the pairs of variables *X*^*j*^ and *Y*^*k*^ with a $\left|M_{k}^{j}\right|$ value greater than the threshold will be aggregated in the Relevance Network. By changing this threshold, the user can choose to include or exclude relationships in the Relevance Network. This option is proposed in an interactive manner in the mixOmics package [12].

Relevance Networks for (r)CCA assume that the underlying network is fully connected, i.e. that there is an edge between any pair of *X* and *Y* variables. For sPLS-reg and sPLS-can, Relevance Networks are solely represented for the variables selected in the model. In this case, $M_{k}^{j}$ pair-wise associations are calculated based on the selected variables.

### Displaying CIM

CIM or heatmaps were introduced in [11,29] to represent data resulting from gene expression profiles. This type of representation is based on a hierarchical clustering simultaneously operating on the rows and columns of a real-valued similarity matrix *M*. The initial matrix is graphically represented as a 2-dimensional coloured image, where each entry of the matrix is coloured on the basis of its value, and where the rows and columns are reordered according to a hierarchical clustering. Dendrograms resulting of the clustering are added to the left (or right) side and to the top (or bottom) of the image. With (r)CCA, (s)PLS-can and (s)PLS-reg, we chose to display CIM based on the pair-wise similarity matrix *M* defined in (1) and in (4).

## Endnotes

^a^http://www.math.univ-toulouse.fr/~biostat/mixOmics

## Competing interests

The authors declare that they have no competing interests.

## Authors contributions

IG performed the statistical analysis, the network analysis, wrote the R functions and drafted the manuscript. KALC performed the statistical analysis and drafted the manuscript. MD performed the some of the network analysis. SD participated in the design of the manuscript and helped to draft the manuscript. All authors read and approved the final manuscript.

## Supplementary Material

Additional file 1 Covariance matrices for the simulated data. Click here for file

Additional file 2 Comparative study of the inferred networks from Pearson correlation, rCCA and PLS-can. Click here for file

Additional file 3 R**script to generate the Relevance Networks for the Nutrimouse and Liver Toxicity data.** Click here for file

Additional file 4 Levels of the clinical chemistry measurements for each group of samples from the hierarchical clustering. Click here for file

Additional file 5 Summary of the 14 networks identified with GeneGo from Liver Toxicity. Click here for file
