# Supplementary material for: Visualising associations between paired ‘omics’ data sets
Source: BioData Min. 2012 Nov 13;5:19. doi: 10.1186/1756-0381-5-19 (PMC3630015; doi:10.1186/1756-0381-5-19)
Supplement: Additional file 1 — Covariance matrices for the simulated data. [file 1756-0381-5-19-S1.pdf]

Simulated data details

For the simulate example we generated 100 X-variables and 50 Y-variables with an equal number of 30 observations in each, such that relevant X and Y variables were generated according to a normal distribution with zero mean and covariance matrix Σ defined by

$$\Sigma = \begin{bmatrix} \Sigma_{XX} & \Sigma_{XY} \\ \\ \Sigma'_{XY} & \Sigma_{YY} \end{bmatrix}, \qquad \Sigma_{XY} = \begin{bmatrix} A_{XY} & 0 & 0 \\ 0 & B_{XY} & 0 \\ 0 & 0 & C_{XY} \end{bmatrix}$$

The matrices that form the basis of these covariances matrices are given below.

Covariance matrix of relevant X variables:

$$\Sigma_{XX} = \begin{bmatrix} A_{XX} & & 0 \\ & B_{XX} & \\ 0 & & C_{XX} \end{bmatrix}$$

matrices A_{XX}, B_{XX} and C_{XX} are given respectively in the tables S1, S2 and S3:

Table S1: A_{XX} covariance matrix.

	X_A^1	X_A^2	X_A^3	X_A^4	X_A^5	X_A^6	X_A^7	X_A^8	X_A^9	X_A^{10}
X_A^1	1.00									
X_A^2	0.85	1.00								
X_A^3	0.87	0.85	1.00							
X_A^4	0.89	0.83	0.84	1.00						
X_A^5	0.87	0.90	0.90	0.90	1.00					
X_A^6	0.90	0.89	0.92	0.89	0.94	1.00				
X_A^7	0.92	0.91	0.93	0.93	0.96	0.95	1.00			
X_A^8	0.89	0.83	0.90	0.94	0.94	0.93	0.96	1.00		
X_A^9	0.86	0.86	0.93	0.90	0.91	0.95	0.96	0.92	1.00	
X_A^{10}	0.82	0.84	0.90	0.86	0.92	0.92	0.95	0.90	0.92	1.00

Table S2: B_{XX} covariance matrix.

	X_B^1	X_B^2	X_B^3	X_B^4	X_B^5	X_B^6	X_B^7	X_B^8	X_B^9	X_B^{10}
X_B^1	1.00									
X_B^2	0.85	1.00								
X_B^3	0.87	0.85	1.00							
X_B^4	0.89	0.83	0.84	1.00						
X_B^5	0.87	0.90	0.90	0.90	1.00					
X_B^6	0.90	0.89	0.92	0.89	0.94	1.00				
X_B^7	0.92	0.91	0.93	0.93	0.96	0.95	1.00			
X_B^8	0.89	0.83	0.90	0.94	0.94	0.93	0.96	1.00		
X_B^8	0.86	0.86	0.93	0.90	0.91	0.95	0.96	0.92	1.00	
X_B^{10}	0.82	0.84	0.90	0.86	0.92	0.92	0.95	0.90	0.92	1.00

Table S3: C_{XX} covariance matrix.

	X_C^1	X_C^2	X_C^3
X_C^1	1.00		
X_C^2	0.94	1.00	
X_C^3	0.96	0.95	1.00

Covariance matrix of relevant Y variables:

$$\Sigma_{YY} = \begin{bmatrix} A_{YY} & & 0 \\ & B_{YY} & \\ 0 & & C_{YY} \end{bmatrix}$$

matrices A_{YY}, B_{YY} and C_{YY} are given respectively in the tables S4, S5 and S6:

Table S4: A_{YY} covariance matrix.

	Y_A^1	Y_A^2	Y_A^3	Y_A^4	Y_A^5	Y_A^6	Y_A^7	Y_A^8	Y_A^9	Y_A^{10}
Y_A^1	1.00									
Y_A^2	0.38	1.00								
Y_A^3	0.44	0.51	1.00							
Y_A^4	0.40	0.55	0.64	1.00						
Y_A^5	0.71	0.62	0.61	0.60	1.00					
Y_A^6	0.54	0.15	0.45	0.47	0.70	1.00				
Y_A^7	0.47	0.63	0.62	0.49	0.73	0.36	1.00			
Y_A^8	0.52	0.50	0.42	0.27	0.73	0.47	0.60	1.00		
Y_A^9	0.56	0.51	0.65	0.49	0.80	0.54	0.57	0.64	1.00	
Y_A^{10}	0.48	0.34	0.39	0.33	0.69	0.49	0.47	0.49	0.65	1.00

Table S5: B_{YY} covariance matrix.

	Y_B^1	Y_B^2	Y_B^3	Y_B^4	Y_B^5
Y_B^1	1.00				
Y_B^2	0.61	1.00			
Y_B^3	0.51	0.32	1.00		
Y_B^4	0.65	0.45	0.60	1.00	
Y_B^5	0.64	0.68	0.54	0.68	1.00

Table S6: C_{YY} covariance matrix.

	Y_C^1	Y_C^2
Y_C^1	1.00	
Y_C^2	-0.96	1.00

Cross-covariance matrix between relevant X and Y variables:

$$\Sigma_{XY} = \begin{bmatrix} A_{XY} & 0 & 0 \\ 0 & B_{XY} & 0 \\ 0 & 0 & C_{XY} \end{bmatrix}$$

matrices A_{XY}, B_{XY} and C_{XY} are given respectively in the tables S7, S8 and S9:

Table S7: A_{XY} covariance matrix.

	X_A^1	X_A^2	X_A^3	X_A^4	X_A^5	X_A^6	X_A^7	X_A^8	X_A^9	X_A^{10}
Y_A^1	-0.72	-0.62	-0.64	-0.70	-0.65	-0.68	-0.71	-0.73	-0.63	-0.67
Y_A^2	-0.60	-0.67	-0.68	-0.66	-0.64	-0.68	-0.70	-0.65	-0.72	-0.64
Y_A^3	-0.61	-0.64	-0.61	-0.73	-0.68	-0.69	-0.69	-0.70	-0.65	-0.65
Y_A^4	-0.54	-0.64	-0.57	-0.59	-0.61	-0.65	-0.59	-0.61	-0.58	-0.56
Y_A^5	-0.88	-0.93	-0.86	-0.88	-0.92	-0.91	-0.93	-0.86	-0.88	-0.87
Y_A^6	-0.62	-0.60	-0.51	-0.61	-0.64	-0.60	-0.62	-0.58	-0.57	-0.56
Y_A^7	-0.61	-0.79	-0.63	-0.67	-0.75	-0.69	-0.71	-0.66	-0.67	-0.64
Y_A^8	-0.71	-0.71	-0.70	-0.69	-0.70	-0.71	-0.74	-0.66	-0.78	-0.73
Y_A^9	-0.84	-0.77	-0.78	-0.82	-0.79	-0.82	-0.82	-0.79	-0.78	-0.72
Y_A^{10}	-0.70	-0.65	-0.64	-0.67	-0.70	-0.66	-0.68	-0.67	-0.63	-0.60

Table S8: B_{XY} covariance matrix.

	X_B^1	X_B^2	X_B^3	X_B^4	X_B^5	X_B^6	X_B^7	X_B^8	X_B^9	X_B^{10}
Y_B^1	0.74	0.78	0.85	0.75	0.81	0.81	0.81	0.78	0.79	0.83
Y_B^2	0.50	0.55	0.61	0.60	0.63	0.60	0.62	0.65	0.60	0.64
Y_B^3	0.68	0.61	0.60	0.63	0.62	0.59	0.65	0.61	0.57	0.53
Y_B^4	0.65	0.63	0.66	0.66	0.66	0.68	0.67	0.68	0.59	0.64
Y_B^5	0.57	0.63	0.62	0.69	0.72	0.65	0.66	0.71	0.64	0.65

Table S9: C_{XY} covariance matrix.

	X_C^1	X_C^2	X_C^3
Y_C^1	0.81	0.81	0.81
Y_C^2	-0.92	-0.91	-0.93
