# Supplementary material for: Visualising associations between paired ‘omics’ data sets
Source: BioData Min. 2012 Nov 13;5:19. doi: 10.1186/1756-0381-5-19 (PMC3630015; doi:10.1186/1756-0381-5-19)
Supplement: Additional file 2 — Comparative study of the inferred networks from Pearson correlation, rCCA and PLS-can. [file 1756-0381-5-19-S2.pdf]

Comparative study of the inferred networks from Pearson correlation, rCCA and PLS-can

We compare the accuracy of the generated networks from Pearson correlation, rCCA and PLS-can with the same simulation setup ('Simulated data' Section and Additional file 1).

Five hundred simulations with 10, 30, 50 and 100 samples were performed. For each simulated X and Y variables, networks were inferred from the first three components of rCCA and PLS-can. The regularization parameters for rCCA were tuned for all the simulations to $\lambda_1 = \lambda_2 = 0.889$.

The error was calculated from "signed" adjacency matrices. Given a network, the (i, j) entry in the matrix is 1 (-1) if there is an edge from the i-th X-variable to the j-th Y-variable with positive (negative) correlation; otherwise the (i, j) entry is 0. If $A = (A_i^j)$ and $B = (B_i^j)$ are the adjacency matrices for networks N_A and N_B, respectively, where N_A and N_B own the same nodes, then the distance between the networks is defined by

$$d(N_A, N_B) = \frac{1}{N} \sum_{i,j=1}^{N} \left| A_i^j - B_i^j \right|$$

where N is the number of nodes in the networks.

The error was calculated as $d(N_{inf}, N_0)$ where N_0 is the inferred network from the initial correlation matrix and N_{inf} is the inferred network from Pearson correlation, rCCA or PLS-can. For a threshold ranging from 0 to 1 with a step of 0.01 errors were averaged over the 500 inferred networks for each threshold value.

Results displayed in Figure S1 show that for a small number of samples, the error rate of the network inferred with Pearson correlation is higher than rCCA and sPLS (up to 10% for a threshold ≤ 0.5 and 10 samples). When the number of samples increases, all networks give similar results for a threshold > 0.4. In the case of a much larger number of variables and for the same number of samples, we would expect the discrepancy between Pearson correlation and rCCA and sPLS to be increased even for a higher threshold.

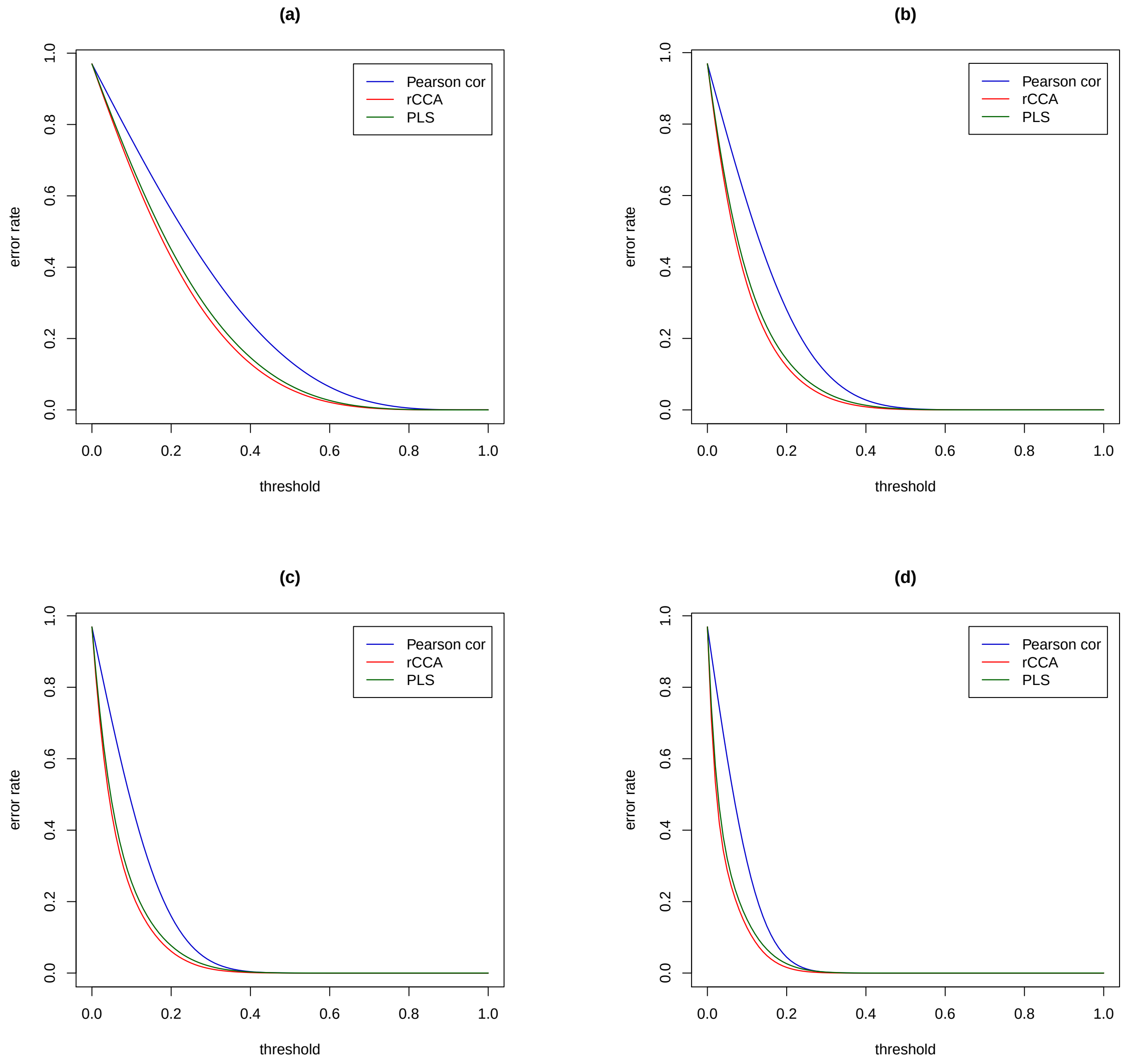

Figure S1: **Error rate for the inferred network from Pearson correlation, rCCA and PLS-can.** Five hundred simulations with 10 (a), 30 (b), 50 (c) and 100 (d) samples.
