# Supplementary material for: Visualising associations between paired ‘omics’ data sets
Source: BioData Min. 2012 Nov 13;5:19. doi: 10.1186/1756-0381-5-19 (PMC3630015; doi:10.1186/1756-0381-5-19)
Supplement: Additional file 3 — Rscript to generate the Relevance Networks for the Nutrimouse and Liver Toxicity data. [file 1756-0381-5-19-S3.pdf]

R Code for the generation of the relevance networks

```
#---------------------------------------------------------------------------#
#-- Importation of the mixOmics package  ----------------------------------#
#---------------------------------------------------------------------------#
library(mixOmics)

#---------------------------------------------------------------------------#
#-- Loading specified data sets / X, Y data matrices definition -----------#
#---------------------------------------------------------------------------#

#-- Nutrimouse data --#
#---------------------#
data(nutrimouse)
X <- nutrimouse$gene
Y <- nutrimouse$lipid

#-- Liver Toxicity data --#
#-------------------------#
data(liver.toxicity)
X <- liver.toxicity$gene
Y <- liver.toxicity$clinic

#---------------------------------------------------------------------------#
#-- Performing Sparse Partial Least Squares  ------------------------------#
#---------------------------------------------------------------------------#
#-- X variables kept in the model on each component
keepX <- rep(50, 3)

#-- Nutrimouse data: canonical mode --#
#-------------------------------------#
```

```
RESULT <- spls(X, Y, ncomp = 3, keepX = keepX, mode = "canonical")


#-- Liver Toxicity data: regression mode --#
#-----------------------------------------#
RESULT <- spls(X, Y, ncomp = 3, keepX = keepX, mode = "regression")


#--------------------------------------------------------------------------#
#-- Generating the relevance networks -------------------------------------#
#--------------------------------------------------------------------------#
relev.net <- network(RESULT, comp = 1:3, threshold = 0.6,
                     color.edge = c("darkgreen", "red"),
                     shape.node = c("rectangle", "circle"),
                     show.color.key = FALSE)


#--------------------------------------------------------------------------#
#-- Exporting graph to Cytoscape file format  -----------------------------#
#--------------------------------------------------------------------------#
write.graph(relev.net$gR, file = "network.gml", format = "gml")
```
