# Supplementary material for: Visualising associations between paired ‘omics’ data sets
Source: BioData Min. 2012 Nov 13;5:19. doi: 10.1186/1756-0381-5-19 (PMC3630015; doi:10.1186/1756-0381-5-19)

Levels of the clinical chemistry measurements on each group of samples for no (A), mild (B) and moderate (C) necrosis

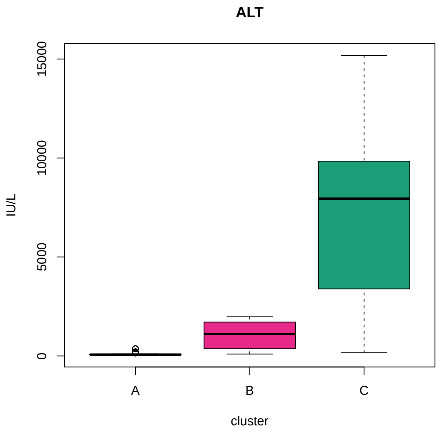

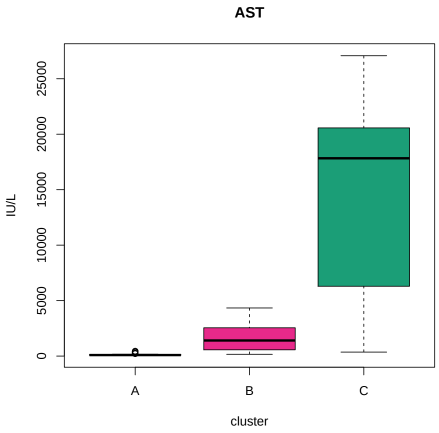

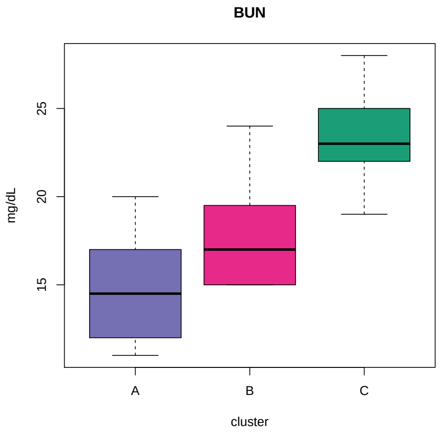

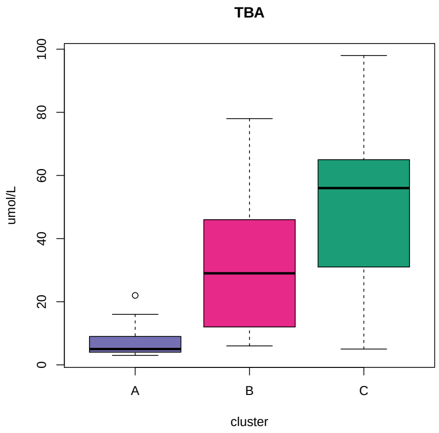

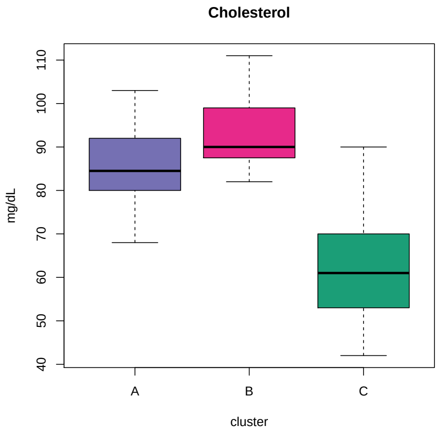

Supplement: Additional file 4 — Levels of the clinical chemistry measurements for each group of samples from the hierarchical clustering. [file 1756-0381-5-19-S4.pdf]
